# Chronic social defeat stress caused region-specific oligodendrogenesis impairment in adolescent mice

**DOI:** 10.3389/fnins.2022.1074631

**Published:** 2023-01-04

**Authors:** Huan Chen, Zhewei Kang, Xueqing Liu, Yinglin Zhao, Zeman Fang, Jinling Zhang, Handi Zhang

**Affiliations:** ^1^Department of Psychiatry, Shantou University Mental Health Center, Shantou, China; ^2^Institute of Mental Health, Peking University Sixth Hospital, Beijing, China

**Keywords:** chronic social defeat stress, adolescent, oligodendrogenesis, oligodendrocyte, oligodendrocyte precursor cell, NG2 cell, proliferation

## Abstract

**Introduction:**

Social stress in adolescents precipitates stress-related emotional disorders. In this study we aimed to investigate oligodendrogenesis in three stress-associated brain regions, medial prefrontal cortex (mPFC), habenula, and amygdala in adolescent mice exposed to social defeat stress.

**Methods:**

Four-week-old adolescent mice were subjected to social defeat for 10 days, followed by behavioral tests and evaluations of oligodendroglial proliferation and differentiation.

**Results:**

Stressed mice showed reduced social interaction, more stretched approach posture, lower sucrose preference, but no changes in the forced swimming test. EdU labeled proliferative cells, newly formed NG2^+^EdU + oligodendrocyte precursor cells (OPCs), and Olig2^+^EdU^+^ oligodendrocyte lineage cells (OLLs) were significantly decreased in the mPFC and the lateral habenula, but not in the amygdala and the medial habenula in socially defeated mice. APC^+^Edu^+^ newly-generated mature oligodendrocytes (OLs) were decreased in the mPFC in stressed mice. However, the total number of NG2^+^ OPCs, APC^+^ mature OLs, and Olig2^+^ OLLs were comparable in all the brain regions examined between stressed and control mice except for a decrease of APC^+^ mature OLs in the prelimbic cortex of stressed mice.

**Conclusion:**

Our findings indicate that adolescent social stress causes emotion-related behavioral changes and region-specific impairment of oligodendrogenesis.

## Introduction

Adolescence is characterized by the first appearance of many psychiatric disorders including stress-related psychiatric disorders (Paus et al., 2008). Social stress such as bullying in adolescence is associated with increased risk of stress-related disorders (Buwalda et al., 2011). However, the underlying mechanism of social stress increasing the risk of stress-related disorders remains unclear. Social defeat in experiment animals is a validated animal model for adolescent social stress, and is suitable to investigate the biological mechanism mediating the influence of social stress (Buwalda et al., 2011).

Oligodendrocytes (OLs) are myelinating cells in the central nervous system. Mature OLs are developed from oligodendrocyte precursor cells (OPCs), which is also known as NG2 cells. OPCs are widely distributed across the brain and able to proliferate and differentiate throughout the whole life. Oligodendrocyte lineage cells (OLLs), including OPCs and mature OLs, play diverse roles in neural plasticity, neuronal nourishing, myelination, and neural repairment, which are important processes for maintaining the brain’s normal physiological and adaptive functions (Sakry and Trotter, 2016; Nave and Werner, 2021; Zhou et al., 2021). Accumulating evidence indicates that the abnormity of OLLs exist in the brain of some psychiatric disorders (Liu et al., 2022), which supports the hypothesis that dysfunction of OLs may be an important pathogenesis mechanism for psychiatric disorders. Additionally, some studies showed that the proliferation and differentiation of OPCs were affected by chronic stress in adult animals (Birey et al., 2015; Yang et al., 2016; Bonnefil et al., 2019; Kokkosis et al., 2022; Poggi et al., 2022), which suggests that the abnormity of OLLs may be an important mechanism mediating the effects of chronic stress on the occurrence of psychiatric disorders. However, the influence of chronic stress on OLLs in the adolescent remains largely unknown.

The medial prefrontal cortex (mPFC), the amygdala (AMY), and the habenula (Hb) are important emotion-related brain regions heavily involved in the biological processes of chronic stress-induced emotional and behavioral alterations. Previous studies have shown that exposure to the chronic stress during adulthood caused the alterations of OLLs-related genes expression in the mPFC, and the abnormity of OLLs may be related to stress-induced emotional behavioral changes (Birey et al., 2015; Yang et al., 2016; Lehmann et al., 2017; Laine et al., 2018; Liu J. et al., 2018; Bonnefil et al., 2019; Cathomas et al., 2019; Kokkosis et al., 2022; Poggi et al., 2022). Adolescence is the key period during which the emotional and cognitive functions rapidly develop and the underlying brain circuits carry out the refinement under the influence of both genetics and experience (Spear, 2000; Meyer and Lee, 2019). Surprisingly, much less is known about the effect of chronic stress on the development of OLLs in these emotion-related cortical and subcortical brain regions in adolescence.

Therefore, in the present study we aimed to investigate the influence of chronic social defeat stress (CSDS) on the generation of OLLs in several emotion-related brain regions, including the mPFC, the AMY, and the Hb, and the emotion-related behavioral changes in adolescent mice.

## Materials and methods

### Animal

Adolescent male C57BL/6JNifdc (aged 3 weeks) mice and adult retired male CD1 breeders (age of 8–10 months) were purchased from Vital River Laboratories (Beijing, China). C57BL/6JNifdc mice were housed in groups of 3–4 in standard polypropylene cages and CD1 retired breeders were individually housed. All animals were housed with a 12-h light-dark cycle (light on at 8:00 a.m.) at controlled temperature (22°C ± 1°C) and were given standard diet and water *ad libitum*. Experiments were conducted in accordance with the guideline of the Animal Care and Use Committee of Shantou University Medical College and approved by the Animal Ethics Committee.

### Experimental protocol

**FIGURE 1:**
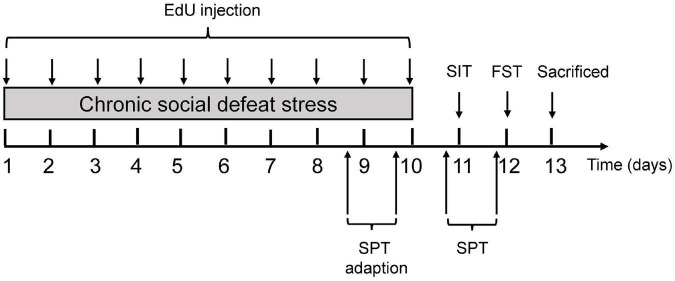
Experimental procedures. Experimental procedure for the chronic social defeat stress (CSDS) paradigm in mice. 5-ethyl-2′-deoxyuridine (EdU) was administered intraperitoneally throughout the 10 days of CSDS to label proliferating cell populations, followed by behavioral tests. Mice were sacrificed for immunofluorescence staining to detect oligodendrocyte pathology after behavioral tests. EdU, 5-ethyl-2′-deoxyuridine; SIT, social interaction test; FST, forced swimming test; SPT, sucrose preference test.

A total of 24 C57 BL/6JNifdc mice were used in our study. After acclimatization for 7 days, C57BL/6JNifdc mice were randomly assigned to either a control or stress group. For randomization, each mouse was assigned a unique identification number at their arrival. We used a computer-generated table of random numbers by Microsoft Excel for randomization, with an identification number of each mouse corresponded to a random number. Mice that corresponded to the smaller random number were assigned to the control group, and the larger random numbers to the stress group. The social defeat stress paradigm was performed as previously described (Golden et al., 2011) with slight modifications. Prior to social defeat stress, CD1 mice were screened for their levels of aggression and those who attacked C57BL/6JNifdc mice within 30 s in two screening tests were chosen for the intruder-resident social defeat stress paradigm. For each stress episode, adolescent C57BL/6JNifdc mice (aged 4 weeks) were introduced into the same compartment of the home cage of the resident CD1 aggressor for 10 min and was physically defeated, and then housed for sensory contact for another 24 h in the compartment adjacent to their respective CD1 aggressor. On the subsequent stress episodes, each intruder C57BL/6JNifdc mouse was introduced to a different CD1 resident every time to ensure the same resident and intruder had never encountered on the consecutive days. This procedure was repeated for ten consecutive days. The mice in the control group were handled at the same time without exposure to a resident aggressor. To minimize the suffering of animals, the experimenters monitored the whole procedure of defeat process all the time. When the experimenters found a C57 BL/6JNifdc mouse was bitten by a CD1 mouse for a prolonged time and could not get free, the mice would be temporarily separated by the experimenter using a wooden strip. The physical status of defeated mice was checked after each defeat episode. Mouse that developed open wounds exceeding 1 cm was removed from the study and given euthanasia. The minor wounds of mice were treated with iodophor diluent to prevent infection. A total of two mice were removed from the study and excluded from the following analysis. As a result, 10 mice in the stress group and 12 mice in the control group were included in the following experiments. Immediately after the last stress episode, both stressed and control mice were individually housed in their home cages under the same conditions. After the social defeat sessions both groups of mice were subjected to behavioral tests and immunochemistry analysis according to the experimental design as shown in Figure 1. Briefly, all the mice were subjected to behavioral tests, including sucrose preference test, social interaction test and forced swimming test in order. One day after the last behavioral test, the mice were sacrificed under deep anesthesia and their brains were processed for immunofluorescence staining to detect OL pathology.

### EdU treatment

For the labeling of proliferating cells, 5-ethyl-2′-deoxyuridine (EdU; Invitrogen, #C10339, Shanghai, China), a thymidine analog that labels dividing cells in the S-phase of the cell cycle, was dissolved in phosphate-buffered saline (PBS; pH7.4) and administrated (5 mg/kg, i.p.) daily before stress episode throughout the 10 days of the CSDS paradigm. The mice in the control group received the same dose of EdU administration as the stress group for 10 days. The dose was selected according to the previous studies (Hill et al., 2014; Dingman et al., 2018) and our preliminary experiment.

### Behavioral tests

#### Social interaction test

Social interaction test (SIT) was performed 24 h after the last stress episode (between 9:00 a.m. and 12:00 p.m.) to determine whether the animals display social avoidance and risk assessment behaviors toward an unfamiliar mouse (social target mouse). As previously established (Golden et al., 2011), social target mice were novel non-aggressive male CD1 mice as determined by pre-screening testing. The test was carried out in an open-field arena (50 cm × 50 cm × 50 cm) with a wire-mesh enclosure (7 cm × 8 cm × 10 cm) put on one side of the field. The test was recorded by a camera above the apparatus. Trajectories of all animals were monitored for the two 2.5-min phases (without or with a CD1 present in the enclosure), separated by a duration of 30 s. The time spent by the experimental mouse in the “interaction zone” (an 8-cm-wide corridor surrounding the enclosure) was analyzed automatically by EthoVision XT 15 (Noldus Information Technology Inc., Beijing, China). We also introduced approaching frequency (frequency of entering the social interaction zone), fleeing frequency (frequency of suddenly retreats from the social interaction zone and runs toward the corners), and stretched approach posture (SAP, characterized by the elongation of the forepart of the animals’ body toward unknown stimuli while the animal keeps a relative safe distance from the possible threat), which are often used to evaluate the approach-avoidance and risk assessment behaviors of mice (Kaesermann, 1986; Henriques-Alves and Queiroz, 2015), and they were manually counted by researchers who were blinded to the group assignment by watching recorded videos.

#### Forced swimming test

Forced swimming test (FST) was used to evaluate behavioral despair in mice. The test was performed between 9:00 a.m. and 12:00 p.m. Mice were placed in a transparent tank [inescapable Plexiglas cylindrical tank (height: 20 cm, diameter: 14 cm)] containing water at a temperature of 25°C (10 cm deep). Each mouse was left to swim for 6 min in the tank, latency to immobility and escape-related mobility behavior during the last 4 min were measured (Porsolt et al., 2001). The mice that acquired an immobile posture, characterized by motionless floating in the water, were termed immobile (immobility time), making only the necessary movements to keep the head above the water. The latency to immobility and immobility time during the last 4 min were analyzed by EthoVision XT 15 (Noldus Information Technology Inc., Beijing, China).

#### Sucrose preference test

The SPT used for evaluating anhedonic behavior was performed as previously described with some modifications (Liu M. Y. et al., 2018). The experiment lasted 96 h, during which all animals were given free access to one bottle of 1% (w/v) sucrose solution and one bottle of regular water, the position of the bottles was switched everyday (8:00 p.m.) to avoid bias for a specific cage side. The first 48 h were the adaptation phase. The second 48 h was the measurement phase. The total liquid intake was comparable between adaption phase and test phase (Supplementary Figure 1). Bottles filled with 1% sucrose solution or water were weighed on the test day (8:00 p.m.) and 12 h later (8:00 a.m.). We finally obtained the consumption of 1% sucrose solution and regular water for two nights. Sucrose preference was expressed as the sucrose solution intake (g)/total fluid intake (g) × 100%. Average sucrose preference or average total fluid consumption over the two nights were analyzed in our study.

### Immunofluorescence staining

The C57BL/6JNifdc mice were anesthetized with isoflurane and perfused intracardially with PBS, followed by 4% paraformaldehyde in PBS. The brains were then removed and fixed in the same fixative overnight at 4°C, and then soaked in 20% sucrose in PBS for 24 h and 30% sucrose in PBS for another 24 h at 4°C. Serial coronal sections (30 μm) were cut on a cryostat (Leica, Wetzlar, Germany). Free-floating sections were washed in PBS, and incubated in a blocking solution composed of 0.3% TritonX-100 and 5% goat serum for 30 min at 22°C. The sections were subsequently incubated with the primary antibody against Olig2 (1:250; Millipore, #AB9610, Shanghai, China), NG2 (1:200; Millipore, # AB5320, Shanghai, China), and APC (1:100; Sigma, #OP80, Shanghai, China), in the blocking solution overnight at 4°C. The above antibodies were used to examine OLLs (anti-Olig2), OPCs (anti-NG2), and mature OLs (anti-APC), respectively. Sections were then rinsed in PBS and incubated in fluorescent secondary antibodies [Goat anti-MS IgG (H + L): 1:1000, Invitrogen, #A28175, Shanghai, China. Goat anti-Rb IgG (H + L): 1:1000, Invitrogen, #A27034, Shanghai, China] for 70 min at 22°C. Sections were then rinsed in PBS and incubated for 10 min with hoechst33342 (1:5000; Invitrogen, #C10339, Shanghai, China). For EdU staining, free-floating sections were washed with 3% bovine serum albumin (BSA) in PBS, and subsequently incubated in EdU reaction cocktail (EdU kit: Invitrogen, #C10339, Shanghai, China) prepared following the manufacturer’s instructions for 30 min at 22°C, followed by three times of rinses in 3% BSA.

### Image analysis

Coronal sections containing the mPFC (Figure 3A), AMY (Figure 5A), and Hb (Figure 7A; Franklin and Paxinos, 2007) were photographed under a Nikon Eclipse-80i microscope (Nikon Instruments Inc., Guangzhou, China). For the analysis of the mPFC, three sections per animal with an interval of 120 μm from bregma +1.98 mm to +1.54 mm were selected for quantitative analysis (Figure 3A). The OLLs in the two subregions of the mPFC, prelimbic cortex (PrL) and infralimbic cortex (IL), were quantified, and the total number of OLLs in the mPFC was also calculated based on the combination of PrL and IL. For the analysis of the AMY, four sections per animal with an interval of 150 μm from bregma −0.94 mm to −1.70 mm were selected for quantification (Figure 5A). The OLLs in the two subregions of AMY, the basolateral amygdala (BLA) and central amygdala (CeA), and in the total AMY areas (BLA+ CeA) were quantified. For the analysis of the Hb, four sections per animal with an interval of 270 μm from bregma −0.94 mm to −2.18 mm were selected (Figure 7A). The OLLs in the two subregions of the Hb, lateral habenular nucleus (LHb) and medial habenular nucleus (MHb), and in the total Hb areas (LHb + MHb) were quantified. Olig2, NG2, APC, and EdU positive cells in the entire area of the selected brain regions were counted by the researchers who were blinded to the group assignment and the results were expressed as the number of positive cells per mm^2^.

### Statistical analysis

We used resource equation method to determine the sample size by the following formula: *E* = Total number of animals-Total number of groups. A total of 12–22 mice are required to keep E between 10 and 20, which is considered as an adequate (Charan and Kantharia, 2013). As death of mice may occur during CSDS, we increased the number to 24, with 12 assigned to the stress group and 12 to the control group. Blinding procedure was performed for data analysis. Firstly, video files of behavioral tests and image files of immunofluorescence experiments were randomly renamed by a researcher using the software “ReNamer.” Secondly, researchers tallied the data of each experiment, i.e., approaching frequency, fleeing frequency, and SAP in the SIT, and cell counts in the immunofluorescence experiments. Finally, data were unblinded and analyzed by appropriate methods for each experiment. All date were reported as mean ± standard error of mean (SEM). Shapiro–Wilk test was used to determine normality of continuous data. For normally distributed data, unpaired *t*-tests were performed to analyze inter-groups differences, and paired sample *t*-test was performed for comparison within groups. For non-normally distributed data, Mann–Whitney *U*-tests were used. Differences were considered statistically significant at *p* < 0.05.

## Results

### Chronic social defeat stress induces emotion-related behavioral changes in adolescent mice

**FIGURE 2:**
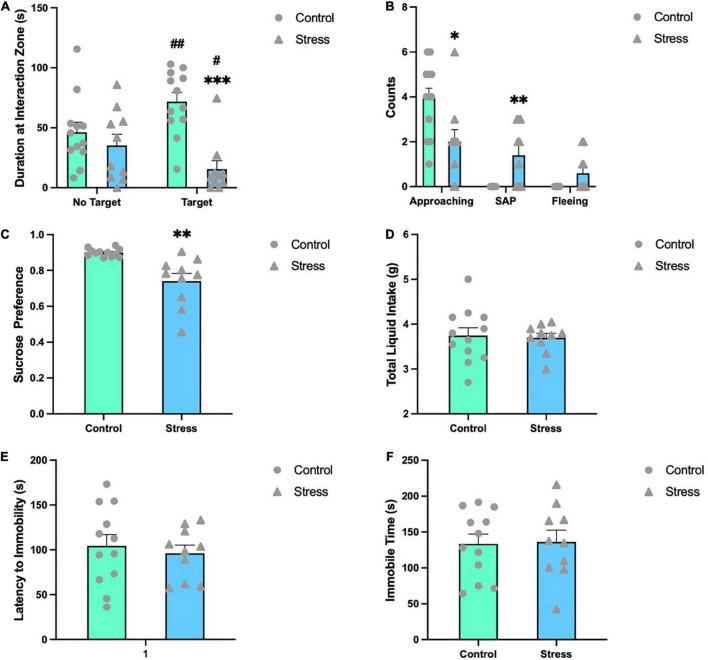
Chronic social defeat stress (CSDS) induces emotion-related behavioral changes in adolescent mice. **(A)** CSDS mice spent significantly less time with a novel social target present in the interaction zone compared to no target present. **(B)** CSDS mice approached less toward the target mouse, exhibited significantly more SAP behaviors and a trend of increase in fleeing frequency than control mice. **(C)** In the sucrose preference test, stressed mice exhibited a significantly less preference to sucrose solution, while the total liquid consumed was comparable between the two groups **(D)**. In the forced swimming test, mice in the two groups showed comparable latency to immobility **(E)** and immobility time **(F)**. Data are expressed as mean ± SEM (Control, *n* = 12; Stress, *n* = 10), *P < 0.05, ^**^P < 0.01, ^***^P < 0.001, Stress vs. Control. ^#^*P* < 0.05, ^##^*P* < 0.01, target vs. no target. SAP, stretched approach posture.

Emotion-related behavioral changes were analyzed shortly after the last stress episode. In SIT, control mice spent significantly more time in the interaction zone with an unfamiliar mouse (target) present than without any mouse (no target) (*t* = −3.941, *P* = 0.002), while stressed mice showed opposite behaviors (*t* = 2.717, *P* = 0.024) (Figure 2A). The time spent with a target mouse present in the interaction zone by control mice was significantly longer than that by stressed mice (*t* = 4.391, *P* < 0.001) (Figure 2A). Furthermore, mice in the CSDS group approached less toward the target mouse (*U* = 25.000, *P* = 0.021), and exhibited significantly more SAP behaviors (*U* = 24.000, *P* = 0.017) and a trend of increase in fleeing frequency (*U* = 36.000, *P* = 0.123) (Figure 2B). In sucrose preference test, CSDS mice developed anhedonia and exhibited a lower sucrose preference than control mice (*t* = 3.960, *P* = 0.001) (Figure 2C), while the total liquid consumed was comparable between the two groups (Figure 2D). In the forced swimming test, neither the latency to immobility (Figure 2E) nor the immobility time (Figure 2F) was different between control and CSDS mice.

**FIGURE 3:**
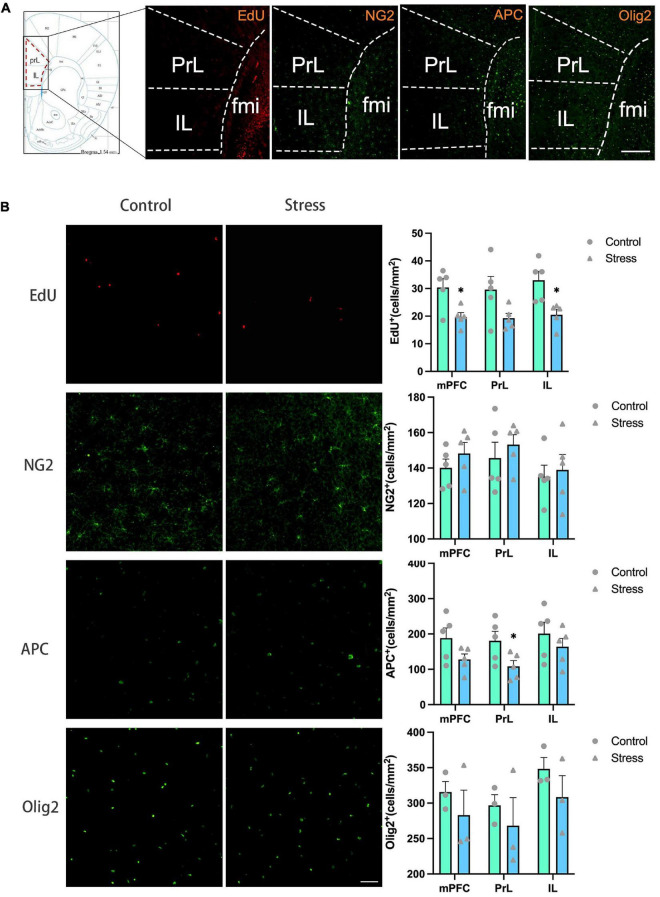
The effects of chronic social defeat stress (CSDS) on the oligodendrocyte lineage cells in the medial prefrontal cortex (mPFC) of adolescent mice. **(A)** Schemata showing the mPFC regions for immunofluorescence and the representative images of immunofluorescence staining of EdU, NG2, APC, and Olig2. Magnification: 100×. Scale bars: 200 μm. **(B)** The representative images of immunofluorescence staining of EdU, NG2, APC, and Olig2 in the mPFC (left panels) and the bar graphs showing the numbers of EdU^+^, NG2^+^, APC^+^, and Olig2^+^ cells in the total mPFC area (PrL + IL), PrL and IL areas (right panels). Magnification: 100×. Scale bars: 50 μm. Data are expressed as mean ± SD. The number of animals used is as follow: EdU (control, *n* = 5; stress, *n* = 5); NG2 (control, *n* = 5; stress, *n* = 5); APC (control, *n* = 5; stress, *n* = 5); Olig2 (control, *n* = 3; stress, *n* = 3), *P < 0.05, Stress vs. Control.

### Chronic social defeat stress reduces oligodendrogenesis in the mPFC

To evaluate the influence of CSDS on oligodendrogenesis, EdU was administrated throughout the CSDS period for the labeling of the proliferative and newly generated cells. OPCs, mature OLs, and total OLLs (including both OPCs and mature OLs) were identified with the markers of NG2, APC, and Olig2, respectively.

The number of EdU^+^ cells was significantly decreased in the total mPFC area (PrL+ IL) (*t* = 2.431, *P* = 0.017) and IL subregion (*t* = 3.416, *P* = 0.010), respectively in adolescent CSDS mice compared to control mice (Figure 3B). The number of NG2^+^ OPCs, APC^+^ mature OLs, and Olig2^+^ OLLs in the IL and the total mPFC areas were comparable between CSDS and control mice, while the number of APC^+^ mature OLs was lower in the PrL in the CSDS mice than that in the control mice (*t* = 2.314, *P* = 0.049) (Figure 3B).

**FIGURE 4:**
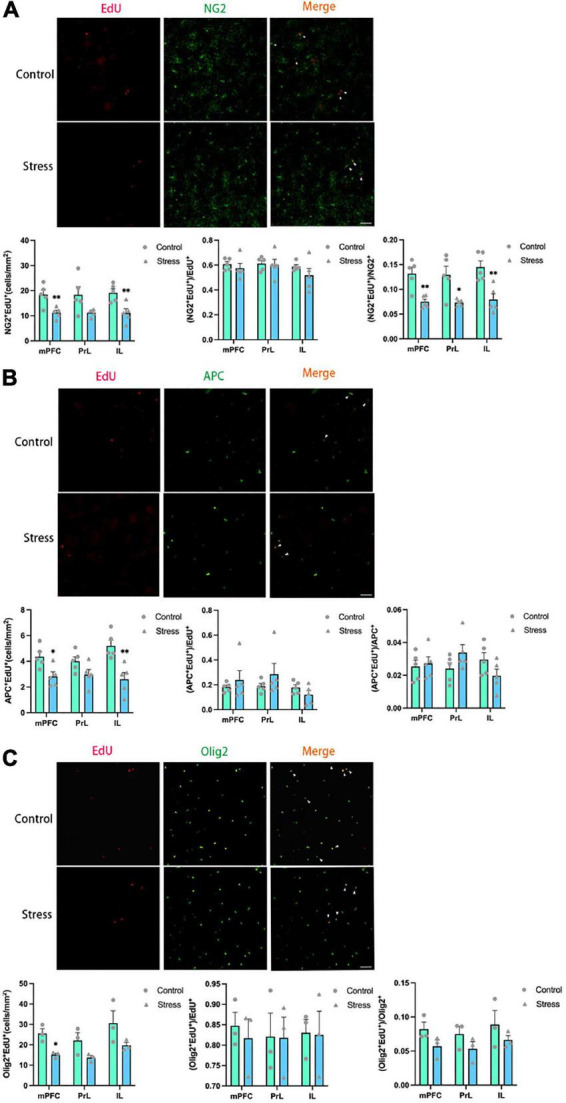
Chronic social defeat stress (CSDS) reduces oligodendrogenesis in the medial prefrontal cortex (mPFC) of adolescent mice. **(A)** Representative images (upper panels) of newly generated cells (EdU^+^; red), OPCs (NG2^+^; green), and newly formed OPCs (NG2^+^EdU^+^; orange) in the mPFC and the bar graphs (lower panels) showing the number of NG2^+^EdU^+^, the percentage of NG2^+^EdU^+^ in the total EdU^+^ cells, and the percentage of NG2^+^EdU^+^ in the total NG2^+^ cells in the total mPFC area and its subregions. The white arrowhead indicates the newly formed OPCs. **(B)** Representative images (upper panels) of newly generated cells (EdU^+^; red), mature OLs (APC^+^; green), and newly formed mature OLs (EdU^+^APC^+^; orange) in the mPFC and the bar graphs (lower panels) showing the number of APC^+^EdU^+^, the percentage of APC^+^EdU^+^ in the total EdU^+^ cells, and the percentage of APC^+^EdU^+^ in the total APC+ cells in the total mPFC area and its subregions. The white arrowhead indicates the newly formed mature OLs. **(C)** Representative images (upper panels) of newly generated cells (EdU^+^; red), OLLs, (Olig2^+^; green), and newly formed OLLs (EdU^+^Olig2^+^; orange) in the mPFC and the bar graphs (lower panels) showing the number of Olig2^+^EdU^+^, the percentage of Olig2^+^EdU^+^ in the total EdU^+^ cells, and the percentage of Olig2^+^EdU^+^ in the total Olig2^+^ cells in the total mPFC area and its subregions. The white arrowhead indicates the newly formed OLLs. Magnification: 100×. Scale bars: 50 μm. Data are expressed as mean ± SEM. The number of animals used is as follow: NG2^+^EdU^+^ (control, *n* = 5; stress, *n* = 5); APC^+^EdU^+^ (control, *n* = 5; stress, *n* = 5); Olig2^+^EdU^+^ (control, *n* = 3; stress, *n* = 3), *P < 0.05, ^**^P < 0.01, Stress vs. Control.

The number of newly formed OPCs identified as NG2^+^Edu^+^ cells (Figure 4A) and newly formed mature OLs identified as APC^+^EdU^+^ cells (Figure 4B) were significantly reduced in the IL (NG2^+^EdU^+^: *t* = 3.519, *P* = 0.008; APC^+^EdU^+^: *t* = 3.805, *P* = 0.005) and the total mPFC areas (NG2^+^EdU^+^: *t* = 3.430, *P* = 0.009; APC^+^EdU^+^: *t* = 2.979, *P* = 0.018) in adolescent CSDS mice. Furthermore, the number of newly developed OLLs identified as Olig2^+^EdU^+^ cells was found to be decreased in the total mPFC area in adolescent CSDS mice (*t* = 4.141, *P* = 0.014) (Figure 4C).

**FIGURE 5:**
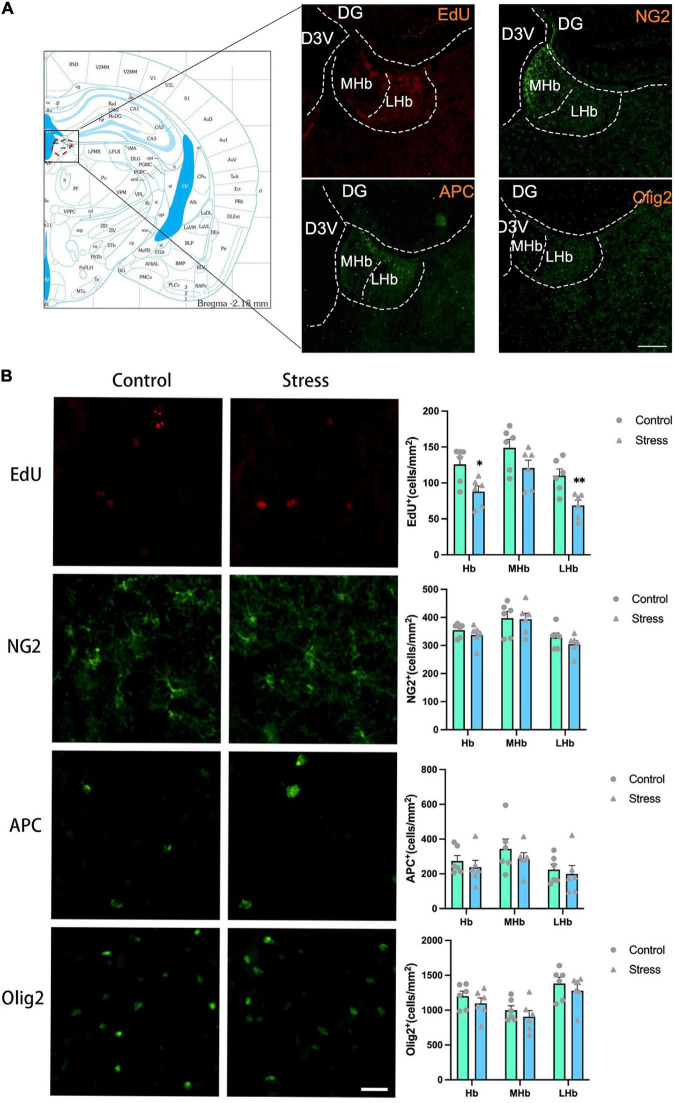
The effects of chronic social defeat stress (CSDS) on the oligodendrocyte lineage cells in the lateral habenula (LHb) of adolescent mice. **(A)** Schemata showing the habenula (Hb) regions for immunofluorescence and the representative images of immunofluorescence staining of EdU, NG2, APC, and Olig2. Magnification: 100×. Scale bars: 200 μm. **(B)** The representative images of immunofluorescence staining of EdU, NG2, APC, and Olig2 in the Hb (left panels) and the bar graphs showing the numbers of EdU^+^, NG2^+^, APC^+^, and Olig2^+^ cells in the total Hb area (LHb + MHb), LHb, and MHb areas (right panels). Magnification: 100×. Scale bars: 25 μm. Data are expressed as mean ± SEM. The number of animals used is as follow: EdU (control, *n* = 6; stress, *n* = 6); NG2 (control, *n* = 6; stress, *n* = 6); APC (control, *n* = 6; stress, *n* = 6); Olig2 (control, *n* = 6; stress, *n* = 6), *P < 0.05, ^**^P < 0.01, Stress vs. Control.

Additionally, the ratios of newly formed OLLs were analyzed. The ratios of newborn OPCs (NG2^+^EdU^+^) to the total NG2^+^ OPCs in all the mPFC regions examined were significantly reduced in adolescent CSDS mice (mPFC: *t* = 4.100, *P* = 0.003; PrL: *t* = 3.136, *P* = 0.014; IL: *t* = 3.835, *P* = 0.005) (Figure 4A). However, the ratios of newly generated mature OLs (APC^+^EdU^+^) to the total APC^+^ mature OLs (Figure 4B) and newly formed OLLs (Olig2^+^EdU^+^) to the total Olig2^+^ OLLs (Figure 4C) were unaltered. Furthermore, the ratios of NG2^+^EdU^+^ OPCs, APC^+^EdU^+^ mature OLs, and Olig2^+^EdU^+^ OLLs to total EdU^+^ cells were unchanged in all the mPFC regions (Figure 4).

### Chronic social defeat stress decreases oligodendrogenesis in the LHb

The exposure to CSDS caused a significant reduction of the number of EdU^+^ cells in the LHb (*t* = 3.555, *P* = 0.005) and the total Hb (LHb + MHb) areas (*t* = 2.970, *P* = 0.014), but not in the MHb in adolescent mice (Figure 5B). The number of NG2^+^ OPCs, APC^+^ mature OLs, and Olig2^+^ OLLs in all the Hb regions were unaltered in adolescent CSDS mice compared to that in control mice (Figure 5B).

**FIGURE 6:**
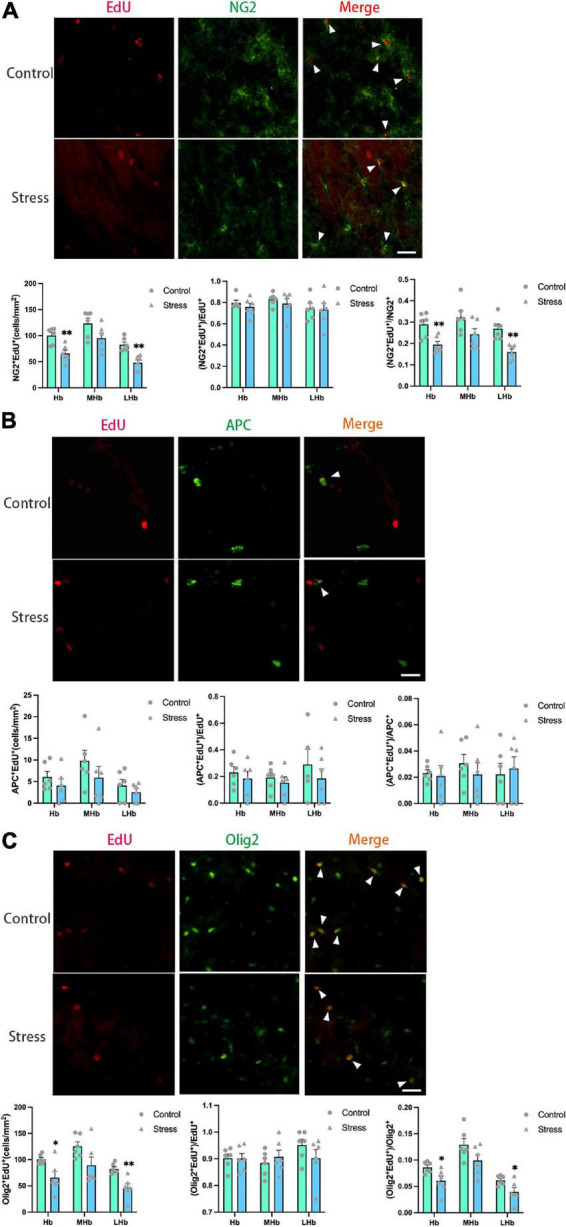
Chronic social defeat stress (CSDS) reduces oligodendrogenesis in the lateral habenula (LHb) of adolescent mice. **(A)** Representative images (upper panels) of newly generated cells (EdU^+^; red), OPCs (NG2^+^; green), and newly formed OPCs (NG2^+^EdU^+^; orange) in the habenula (Hb) and the bar graphs (lower panels) showing the number of NG2^+^EdU^+^, the percentage of NG2^+^EdU^+^ in the total EdU^+^ cells, and the percentage of NG2^+^EdU^+^ in the total NG2^+^ cells in the total Hb area and its subregions. The white arrowhead indicates the newly formed OPCs. **(B)** Representative images (upper panels) of newly generated cells (EdU^+^; red), mature OLs (APC^+^; green), and newly formed mature OLs (EdU^+^APC^+^; orange) in the Hb and the bar graphs (lower panels) showing the number of APC^+^EdU^+^, the percentage of APC^+^EdU^+^ in the total EdU^+^ cells, and the percentage of APC^+^EdU^+^ in the total APC^+^ cells in the total Hb area and its subregions. The white arrowhead indicates the newly formed mature OLs. **(C)** Representative images (upper panels) of newly generated cells (EdU^+^; red), OLLs, (Olig2^+^; green), and newly formed OLLs (EdU^+^Olig2^+^; orange) in the Hb and the bar graphs (lower panels) showing the number of Olig2^+^EdU^+^, the percentage of Olig2^+^EdU^+^ in the total EdU^+^ cells, and the percentage of Olig2^+^EdU^+^ in the total Olig2^+^ cells in the total Hb area and its subregions. The white arrowhead indicates the newly formed OLLs. Magnification: 100×. Scale bars: 25 μm. Data are expressed as mean ± SEM. The number of animals used is as follow: NG2^+^EdU^+^ (control, *n* = 6; stress, *n* = 6); APC^+^EdU^+^ (control, *n* = 6; stress, *n* = 6); Olig2^+^EdU^+^ (control, *n* = 6; stress, *n* = 6). *P < 0.05, ^**^P < 0.01, Stress vs. Control.

The numbers of newly formed OPCs (NG2^+^Edu^+^) (Figure 6A) and newly developed OLLs (Olig2^+^EdU^+^) (Figure 6C) in the LHb (NG2^+^EdU^+^: *t* = 4.389, *P* = 0.001; Olig2^+^EdU^+^: *t* = 4.011, *P* = 0.002) and the total Hb areas (NG2^+^EdU^+^: *t* = 3.753, *P* = 0.004; Olig2^+^EdU^+^: *t* = 2.800, *P* = 0.019) were lower in CSDS mice than that in control mice. No difference was found on the number of newly formed mature OLs (APC^+^EdU^+^ cells) in all the Hb areas between stressed and control mice (Figure 6B).

**FIGURE 7:**
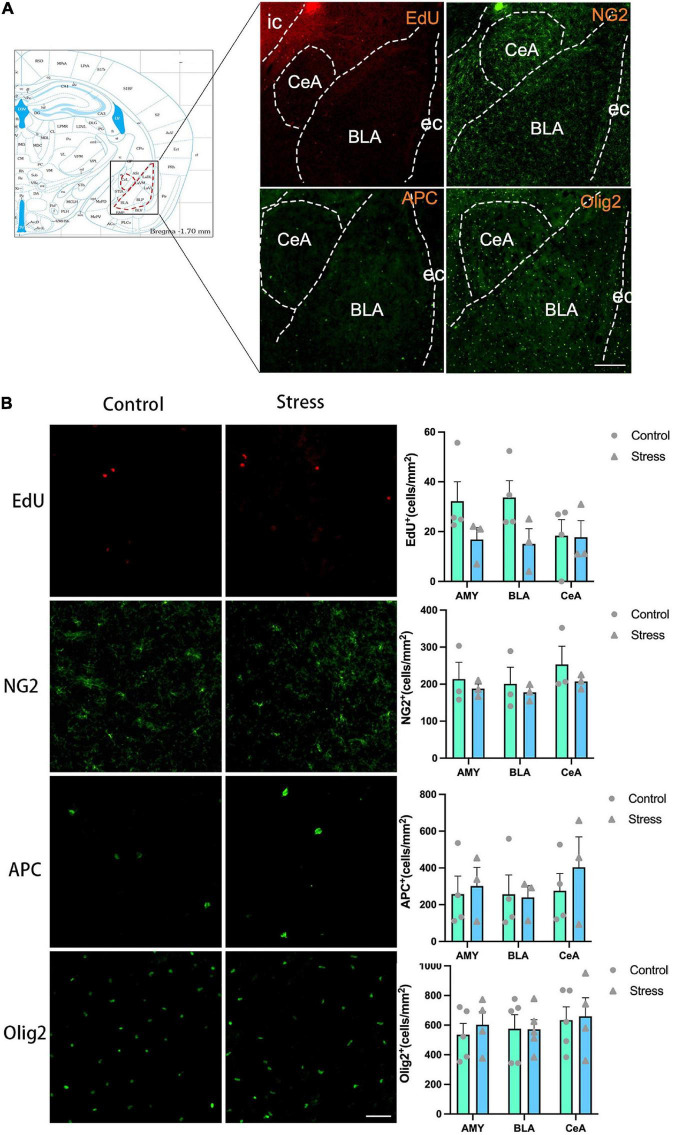
The effects of chronic social defeat stress (CSDS) on the oligodendrocyte lineage cells in the amygdala (AMY) of adolescent mice. **(A)** Schemata showing the AMY regions for immunofluorescence and the representative images of immunofluorescence staining of EdU, NG2, APC, and Olig2. Magnification: 100×. Scale bars: 200 μm. **(B)** The representative images of immunofluorescence staining of EdU, NG2, APC, and Olig2 in the AMY (left panels) and the bar graphs showing the numbers of EdU^+^, NG2^+^, APC^+^, and Olig2^+^ cells in the total AMY area (BLA + CeA), BLA and CeA areas (right panels). Magnification: 100×. Scale bars: 50 μm. Data are expressed as mean ± SEM. The number of animals used is as follow: EdU (control, *n* = 4; stress, *n* = 3); NG2 (control, *n* = 3; stress, *n* = 3); APC (control, *n* = 4; stress, *n* = 3); Olig2 (control, *n* = 5; stress, *n* = 4), Stress vs. Control.

Additionally, the proportions of newborn OPCs (NG2^+^EdU^+^) in the total NG2^+^ OPCs (Figure 6A) and newly developed OLLs (Olig2^+^EdU^+^) in the total Olig2^+^ OLLs (Figure 6C) were significantly reduced in the LHb (NG2^+^EdU^+^/NG2^+^: *t* = 4.234, *P* = 0.002; Olig2^+^EdU^+^/Olig2^+^: *t* = 2.408, *P* = 0.037) and the total Hb areas (NG2^+^EdU^+^/NG2^+^: *t* = 3.484, *P* = 0.006; Olig2^+^EdU^+^/Olig2^+^: *t* = 2.527, *P* = 0.030) of CSDS mice compared to that of the control mice. However, the proportion of newly produced APC^+^EdU^+^ mature OLs in the total APC+ mature OLs were comparable between the two groups (Figure 6B). Furthermore, the proportions of NG2^+^EdU^+^ OPCs, APC^+^EdU^+^ mature OLs, and Olig2^+^EdU^+^ OLLs in the total EdU^+^ cells were unchanged in all the Hb regions (Figure 6).

### Chronic social defeat stress exerts little effect on oligodendrogenesis in the AMY

**FIGURE 8:**
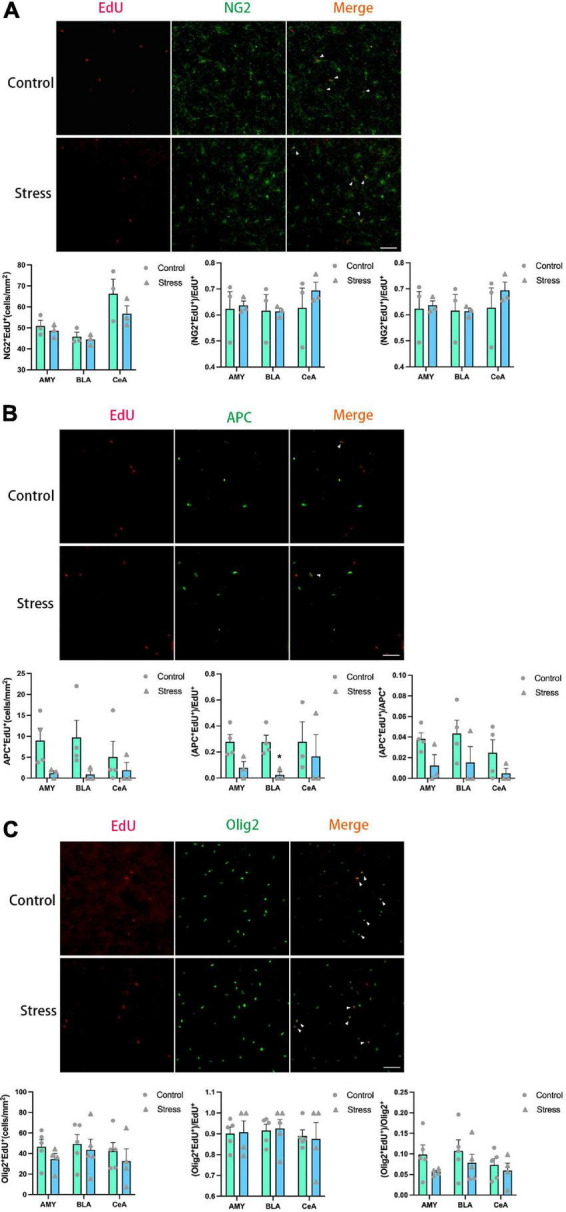
Chronic social defeat stress (CSDS) exerts little effects on oligodendrogenesis in the amygdala (AMY) of adolescent mice. **(A)** Representative images (upper panels) of newly generated cells (EdU^+^; red), OPCs (NG2^+^; green), and newly formed OPCs (NG2^+^EdU^+^; orange) in the AMY and the bar graphs (lower panels) showing the number of NG2^+^EdU^+^, the percentage of NG2^+^EdU^+^ in the total EdU^+^ cells, and the percentage of NG2^+^EdU^+^ in the total NG2^+^ cells in the total AMY area and its subregions. The white arrowhead indicates the newly formed OPCs. **(B)** Representative images (upper panels) of newly generated cells (EdU^+^; red), mature OLs (APC^+^; green), and newly formed mature OLs (EdU^+^APC^+^; orange) in the AMY and the bar graphs (lower panels) showing the number of APC^+^EdU^+^, the percentage of APC^+^EdU^+^ in the total EdU^+^ cells, and the percentage of APC^+^EdU^+^ in the total APC + cells in the total AMY area and its subregions. The white arrowhead indicates the newly formed mature OLs. **(C)** Representative images (upper panels) of newly generated cells (EdU^+^; red), OLLs, (Olig2^+^; green), and newly formed OLLs (EdU^+^Olig2^+^; orange) in the AMY and the bar graphs (lower panels) showing the number of Olig2 + EdU+, the percentage of Olig2 + EdU+ in the total EdU + cells, and the percentage of Olig2^+^EdU^+^ in the total Olig2^+^ cells in the total AMY area and its subregions. The white arrowhead indicates the newly formed OLLs. Magnification: 100×. Scale bars: 50 μm. Data are expressed as mean ± SEM. The number of animals used is as follow: NG2^+^EdU^+^ (control, *n* = 3; stress, *n* = 3); APC^+^EdU^+^ (control, *n* = 4; stress, *n* = 3); Olig2^+^EdU^+^ (control, *n* = 5; stress, *n* = 4). *P < 0.05, Stress vs. Control.

The experience of CSDS did not affect the numbers of EdU^+^ cells, NG2^+^ OPCs, APC^+^ mature OLs, and Olig2^+^ OLLs in the AMY and its subregions (Figure 7B). Furthermore, the number of the newly formed OPCs (NG2^+^EdU^+^), OLs (APC^+^EdU^+^), and OLLs (Olig2^+^EdU^+^) was not changed in all the areas of amygdala in adolescent mice (Figure 8). Only the percentage of EdU^+^APC^+^ mature OLs in the total EdU^+^ cells were decreased in the BLA in CSDS mice (*t* = 3.790, *P* = 0.013) (Figure 8B), while the percentages of other newly formed OLLs in the total EdU^+^ cells were not altered (Figure 8).

## Discussions

In the present study, we investigated the effect of adolescent chronic social defeat on the emotion-related behavioral changes and the development of OLLs in mouse mPFC, AMY, and Hb. We demonstrated that CSDS caused social avoidance and anhedonia and affected the proliferation and maturation of OLLs in a region-specific manner in adolescent mice. A summary of OLLs findings is presented in Table 1.

**TABLE 1:** The summary of the effect of CSDS on oligodendrogenesis in the mPFC, Hb, and AMY.

	mPFC			Hb			AMY		
	**PrL + IL**	**PrL**	**IL**	**LHb + MHb**	**LHb**	**MHb**	**BLA + CeA**	**BLA**	**CeA**
EdU^+^	↓	–	↓	↓	↓	–	–	–	–
NG2^+^	–	–	–	–	–	–	–	–	–
NG2^+^EdU^+^	↓	–	↓	↓	↓	–	–	–	–
NG2^+^EdU^+^/EdU^+^	–	–	–	–	–	–	–	–	–
NG2^+^EdU^+^/NG2^+^	↓	↓	↓	↓	↓	–	–	–	–
APC^+^	–	↓	–	–	–	–	–	–	–
APC^+^EdU^+^	↓	–	↓	–	–	–	–	–	–
APC^+^EdU^+^/EdU^+^	–	–	–	–	–	–	–	↓	–
APC^+^EdU^+^/APC^+^	–	–	–	–	–	–	–	–	–
Olig2^+^	–	–	–	–	–	–	–	–	–
Olig2^+^EdU^+^	↓	–	–	↓	↓	–	–	–	–
Olig2^+^EdU^+^/EdU^+^	–	–	–	–	–	–	–	–	–
Olig2^+^EdU^+^/Olig2^+^	–	–	–	↓	↓	–	–	–	–

mPFC, medial prefrontal cortex; Hb, habenula; AMY, amygdala; PrL, prelimbic; IL, infralimbic; LHb, lateral habenula; MHb, medial habenula; BLA, basolateral amygdala; CeA, central amygdala.

Our data showed that CSDS caused emotion-related behavioral changes including social avoidance, increased risk assessment behavior (SAP), and lower sucrose preference (anhedonia) in adolescent mice, while the immobility time in the forced swimming test was unaffected. These behavioral changes induced by CSDS largely agree with the previous studies using adolescent (Huang et al., 2013; Iñiguez et al., 2014; Mouri et al., 2018; Alves-Dos-Santos et al., 2020; Shimizu et al., 2020) or adult animals (Krishnan et al., 2007). The effects of CSDS on the forced swimming test in adolescent mice were mixed. Some studies showed increased immobility time in stressed mice (Huang et al., 2013; Iñiguez et al., 2014; Shimizu et al., 2020), while others found no alterations (Mouri et al., 2018). This discrepancy may be related to the difference on the age of mice used in these studies, since the rapid developmental changes of brain with time during adolescence may result in different behavioral responses to the stress. These results indicate that CSDS may cause different combinations of emotion-related behaviors which may depend on the timing of stress exposure in adolescent animals.

The previous studies investigating the effect of chronic stress on OLLs mainly focused on the adult animals, while much less studies took use of adolescent animals. Our results showed that CSDS resulted in the significant decrease of the number of proliferative cells (EdU^+^ cells), newly born OLLs including OPCs and mature OLs in the mPFC of adolescent animals, while it had little effect on the number of preexisting OLLs. The reduction of proliferative cells in the mPFC have been observed in the adult rats exposed to chronic stress in the previous studies (Banasr et al., 2007; Czéh et al., 2007), and our result extends these findings to the adolescent animals. The majority of EdU^+^ cells in the mPFC are OLLs in the current study (around 80% as shown in Figure 4), which is consistent with the previous reports using adult animals (Banasr et al., 2007; Czéh et al., 2007). Other types of cells are majorly endothelial cells which occupy a small portion of the total EdU^+^ cells (Czéh et al., 2007). In the present study, although the number of newly generated OLLs decreased after exposure to CSDS, the ratios of newly generated OLLs to the total EdU^+^ cells were not altered. These results indicated that the effect of CSDS on EdU^+^ cells in the mPFC is not specific to newly OLLs.

The total number of OPCs (NG2^+^ cells) was not changed, while the number of newborn OPCs (NG2^+^EdU^+^) was reduced in the mPFC of adolescent CSDS mice. The result suggests that CSDS mainly affect the OPCs in the active proliferative status, but not the OPCs in a quiescent status, and the proliferative process of OPCs may be inhibited by CSDS. Since the proportion of newborn OPCs (NG2^+^EdU^+^ cells) in the total OPCs in the mPFC was very low (lower than 10% as shown in Figure 4), the decrease of this cellular population may not significantly influence the total number of OPCs population. Similarly, we found exposure to CSDS caused a significant reduction of the number of newly generated mature OLs (APC^+^EdU^+^ cells) in the mPFC, but not the total number of APC^+^ mature OLs. One explanation to this phenomenon is that the less production of newborn OPCs caused by CSDS in adolescent mice consequently leads to the less development of new OLs. An alternative explanation is that the differentiation of newborn OPCs into mature OLs might be compromised by the CSDS. Additionally, the proportion of newly generated mature OLs (APC^+^EdU^+^ cells) in the total APC+ mature OLs is also very low (around 3% as shown in Figure 4), the decrease of this cellular population may not significantly influence the total number of mature APC^+^ OLs. Furthermore, we found in the CSDS mice the number of Olig2^+^ OLLs was unaltered, while the number of newly produced OLLs (Olig2^+^EdU^+^ cells) was significantly reduced, and the ratio of Olig2^+^EdU^+^ OLLs to the total EdU^+^ cells was unchanged. These results further confirmed our findings that newly generated OLLs were reduced in the mPFC in adolescent CSDS animals.

Recent studies have demonstrated that the production of new OLLs in the brain was required for motor-skill learning (McKenzie et al., 2014; Xiao et al., 2016), which suggests that generating new OLLs in the brain is an important mechanism of neural plasticity which contributes to the behavioral adaption to the environment. Evidences also indicated that short-term stress exposure, which majorly induced adaptive behavioral changes, resulted in an increase of NG2 cell number in the mPFC, whereas long-term stress exposure, which usually resulted in maladaptive behaviors, caused a decrease of NG2 cell number (Birey et al., 2015). These evidences suggest that newborn OLLs, despite occupying a small proportion of the whole cell population in the mPFC, may play a key role in the maturation and plasticity of frontal neural circuits mediating the emotional and cognitive functions. It is plausible that CSDS may inhibit the generation of new OLLs in adolescent animals, thus result in the impairment of plasticity of frontal neural circuit, and affect the adaption of the animals to the environmental stimulus and finally develop a maladaptive behavioral phenotype.

The Hb consists of two anatomically and functionally discrete nuclei, the LHb and the MHb, which is enriched in myelinated neuronal fibers and serves as a central structure connecting forebrain to midbrain regions (Yoo et al., 2020). Recently, the Hb has emerged as a new player in regulating the process of rewarding, emotion, and stress reaction. Evidence has accumulated to show that Hb dysfunction is associated with the development of psychiatric disorders, such as depression, addition, and schizophrenia (Boulos et al., 2017). To our knowledge, the current study is the first to explore the effect of chronic stress on OLLs in the Hb. We found that CSDS mainly disrupted the homeostasis of OLLs development in the LHb, while the proliferation and maturation of OLLs in the MHb were largely undisturbed. This result is consistent with previous studies showing that the LHb dysfunction, but not the MHb, was implicated in the pathophysiology of depression (Browne et al., 2018). Analogous to the mPFC, CSDS significantly decreased the number of proliferative cells (EdU^+^ cells) and newly generated OLLs (NG2^+^EdU^+^, APC^+^EdU^+^, and Olig2^+^EdU^+^ cells) in the LHb in the adolescent mice, while it had little effect on the number of the preexisting OLLs (NG2^+^, APC^+^, and Olig2^+^ cells) and the proportions of these OLLs in the total EdU^+^ cells. These data indicates that proliferative cells and newly produced OLLs may be more sensitive to the destructive effect of chronic stress than the preexisting OLLs. The dysfunction of the LHb OLLs may be one of the underlying mechanisms mediating the maladaptive behaviors induced by CSDS in adolescent mice.

The amygdala is composed of clustered heterogeneous subnuclei, which is important for emotion processing and approach-avoidance behaviors (Schumann et al., 2011). Our data indicated that CSDS had little effect on the number of proliferative cells (EdU^+^ cells), OLLs (NG2^+^, APC^+^, and Olig2^+^ cells), and newly generated OLLs (NG2^+^EdU^+^, APC^+^EdU+, and Olig2^+^EdU+ cells) in the AMY and its subnuclei (BLA and CeA) in the adolescent mice. Consistent with our observation on proliferative cells, one previous study showed that in adolescent rats 3-day repeated variable stress caused no alteration on the number of bromodeoxyuridine (BrdU)-labeled newly generated cells in the whole AMY 3 days after the end of stress (Saul et al., 2015). However, in the same study newly produced NG2 cells in the AMY was significantly decreased when examined 12 days after the end of stress exposure (Saul et al., 2015), which suggests a delayed impairment on OLs proliferation in the AMY in adolescent animals. We observed a decrease on the ratio of newly generated mature OLs (APC^+^EdU^+^) to the total EdU^+^ cells only in the BLA, which indicates that the differentiation of newborn OPCs into mature OLs might be impaired by CSDS specifically in this region. Notably, another study in adult mice found that a 15-day social defeat stress led to a reduction in proliferative OPCs and an increment in OLs in the BLA (Poggi et al., 2022), which indicates that stress exposure may accelerate the differentiation of OPC in the BLA in adult animals. These data suggest that stress exposure may lead to a development-dependent dynamic change in OLLs proliferation and maturation in the AMY, while the exact mechanisms underlying the discrepancy among these studies remains unclear and are needed to be clarified in the future study.

In summary, our results show that CSDS leads to emotion-related behavioral changes in adolescent mice, which is accompanied by a region-specific impairment of oligodendrogenesis in the mPFC and LHb. These results suggest that the deficit of OL plasticity might be one of the key pathogenesis underlying stress-related psychiatric disorders.

## Data availability statement

The raw data supporting the conclusions of this article will be made available by the authors, without undue reservation.

## Ethics statement

The animal study was reviewed and approved by the Animal Ethics Committee of Shantou University Medical College.

## Author contributions

HZ, JZ, and YZ contributed to the conception and design of the study. HC, ZK, XL, and ZF performed the behavioral tests. HC and ZK conducted the immunofluorescence experiments and data analysis and prepared the draft of the manuscript. All authors contributed to the data interpretation, manuscript read and revision, and approved the submitted version.
