# Supplementary material for: Chronic social defeat stress caused region-specific oligodendrogenesis impairment in adolescent mice
Source: Front Neurosci. 2023 Jan 4;16:1074631. doi: 10.3389/fnins.2022.1074631 (PMC9846137; doi:10.3389/fnins.2022.1074631)
Supplement: Supplementary file 1 [file Image_1.pdf]

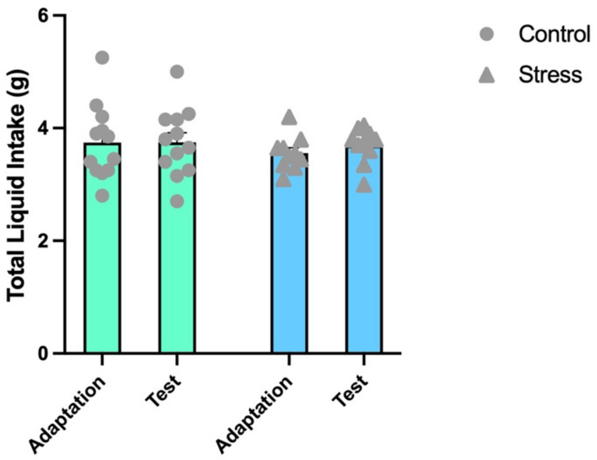

Figure 1S. The total liquid intake was comparable between adaptation and test phase of sucrose preference test. No significant difference of total liquid intake was found in both control (Adaptation vs. Test, t = -3.74, p = 0.716) or stress (Adaptation vs. Test, t = -1.510, p = 0.165) group. The average intake of total liquid over two nights (8 pm to 8 am) was used for analysis. Data are expressed as mean ± SEM (Control, n = 12; Stress, n = 10).
